# ADHD-related sex differences in fronto-subcortical intrinsic functional connectivity and associations with delay discounting

**DOI:** 10.1186/s11689-018-9254-9

**Published:** 2018-12-13

**Authors:** Keri S. Rosch, Stewart H. Mostofsky, Mary Beth Nebel

**Affiliations:** 1 Center for Neurodevelopmental and Imaging Research, Kennedy Krieger Institute, Baltimore, MD 21205 USA; 2 Department of Neuropsychology, Kennedy Krieger Institute, Baltimore, MD 21205 USA; 3 Department of Psychiatry and Behavioral Sciences, Johns Hopkins University School of Medicine, Baltimore, MD 21205 USA; 4 Department of Neurology, Johns Hopkins University School of Medicine, Baltimore, MD USA

**Keywords:** ADHD, fMRI, Reward, Delay discounting, Temporal discounting, Resting-state, Functional connectivity, ICA

## Abstract

**Background:**

Attention-deficit/hyperactivity disorder (ADHD) is associated with atypical fronto-subcortical neural circuitry and heightened delay discounting, or a stronger preference for smaller, immediate rewards over larger, delayed rewards. Recent evidence of ADHD-related sex differences in brain structure and function suggests anomalies in fronto-subcortical circuitry may differ among girls and boys with ADHD. The current study examined whether the functional connectivity (FC) within fronto-subcortical neural circuitry differs among girls and boys with ADHD compared to same-sex typically developing (TD) controls and relates to delay discounting.

**Methods:**

Participants include 8–12-year-old children with ADHD (*n* = 72, 20 girls) and TD controls (*n* = 75, 21 girls). Fronto-subcortical regions of interest were functionally defined by applying independent component analysis to resting-state fMRI data. Intrinsic FC between subcortical components, including the striatum and amygdala, and prefrontal components, including ventromedial prefrontal cortex (vmPFC), anterior cingulate cortex (ACC), and anterior dorsolateral prefrontal cortex (dlPFC), was compared across diagnostic groups overall and within sex. Correlations between intrinsic FC of the six fronto-subcortical pairs and delay discounting were also examined.

**Results:**

Both girls and boys with ADHD show atypical FC between vmPFC and subcortical regions including the striatum (stronger positive FC in ADHD) and amygdala (weaker negative FC in ADHD), with the greatest diagnostic effects among girls. In addition, girls with ADHD show atypical intrinsic FC between the striatum and dlPFC components, including stronger positive FC with ACC and stronger negative FC with dlPFC. Further, girls but not boys, with ADHD, show heightened real-time delay discounting. Brain–behavior correlations suggest (1) stronger negative FC between the striatal and dlPFC components correlated with greater money delay discounting across all participants and (2) stronger FC between the amygdala with both the dlPFC and ACC components was differentially related to heightened real-time discounting among girls and boys with and without ADHD.

**Conclusions:**

Our findings suggest fronto-subcortical functional networks are affected in children with ADHD, particularly girls, and relate to delay discounting. These results also provide preliminary evidence of greater disruptions in fronto-subcortical FC among girls with ADHD that is not due to elevated inattention symptom severity, intellectual reasoning ability, age, or head motion.

**Electronic supplementary material:**

The online version of this article (10.1186/s11689-018-9254-9) contains supplementary material, which is available to authorized users.

## Introduction

Attention-deficit/hyperactivity disorder (ADHD) is a neurodevelopmental disorder characterized by developmentally inappropriate and impairing inattention, hyperactive, and impulsive behaviors. Etiological models of ADHD postulate dysfunction in fronto-subcortical neural pathways involved in executive functions and motivation as contributing to deficient self-regulation of cognition, behavior, and emotion [1, 2]. Executive function (EF) refers to the deliberate, top–down control of thoughts, actions, and emotions in the service of goal-directed behavior [3] and is generally purported to rely on discrete cortico-striatal-thalamo-cortical loops [4–9]. Cognition and motivation and the associated neural circuitry interact to produce adaptive and maladaptive behavior [10]. The interaction of cognition and motivation guides reward-based decision-making in the form of delay discounting, a well-established phenomenon in which individuals discount the value of a reward as a function of delay to receiving the reward [11, 12]. The ability to inhibit a response to an immediately available reward in pursuit of a larger or more valuable, albeit delayed, reward is a critical component of cognitive, emotional, and social development. Failure to inhibit such a response is thought to be a central feature of pathological behavior associated with impulsivity including ADHD, substance abuse, obesity, and gambling [13–15].

Delay discounting is central to many theories of ADHD, which postulate altered reinforcement sensitivity [16] either due to attenuation of dopamine signaling to delayed reward [17], a failure of anticipatory dopamine cell firing [18], or a breakdown in higher order control resulting in an inability to suppress the drive (i.e., resist temptation) to respond to the immediate option [19]. Although delay discounting is typically described as reflecting reward sensitivity, there is growing evidence that delay aversion may also contribute to one’s preference for immediate over delayed rewards. Sonuga-Barke and colleagues proposed that delay is an aversive experience in and of itself, eliciting a negative affective state, which children with ADHD work to escape or avoid [20, 21]. Delay aversion may also work in concert with an impulsive drive for immediate reward to exacerbate impulsive choice [1, 22]. Neuroimaging research has implicated fronto-subcortical circuitry in delay discounting as part of a cognitive control network including the dorsolateral PFC (dlPFC) and anterior cingulate cortex (ACC) and a reward valuation network including the ventromedial (vmPFC)/orbitofrontal cortex (OFC) and ventral striatum (VS)/nucleus accumbens (NAcc) [11]. In addition, task-based fMRI studies have shown involvement of the amygdala in delay discounting among individuals with ADHD [23, 24], in support of the delay aversion theory of ADHD. Thus, variability in fronto-subcortical neural circuitry implicated in ADHD may be associated with individual differences in delay discounting.

Evidence of ADHD-associated disruptions in intrinsic fronto-subcortical functional connectivity (FC) using resting state functional MRI (rs-fMRI) has been inconsistent. In general, studies have shown aberrant FC of fronto-subcortical networks in children and adolescents with ADHD (see reviews by [25, 26]). However, the specific regions involved and whether a group effect or an association with ADHD symptoms was observed and the direction of the observed group effect or symptom association have all varied [27–29]. Studies examining striatum-vmPFC FC have reported greater FC [30–32] and similar FC among children and adolescents with ADHD compared to controls [33]. In contrast, studies of striatal-dlPFC FC have reported weaker FC with the VS [34], dorsal caudate [33], and putamen [35] in children and adolescents with ADHD. Further, findings from the same researchers among a sample of partially overlapping participants reported both stronger [31] and weaker NAcc-anterior PFC FC in ADHD [32], possibly due to the different methods used to define the NAcc seed region or a more heterogeneous ADHD sample in the latter study. Finally, two studies examining a much wider age range (e.g., 8–30 years) did not find evidence of aberrant cortico-striatal networks in ADHD [27, 29]. Only one study to date has examined associations between rs-fMRI FC and delay discounting in children with ADHD, reporting that increased NAcc-anterior PFC FC in ADHD positively correlated with delay discounting [31]. While the majority of studies in the ADHD literature have used seed-based analyses, they have varied in their selection and definition of the seed regions. We chose to apply a combined data- and hypothesis-driven approach in which we use group-independent components analyses (ICA) to identify the intrinsic functional networks rather than defining seed-regions based on anatomical boundaries or as spheres centered around reported peaks of task activation with arbitrary radii. To focus our analyses on fronto-subcortical regions, we selected components with the greatest spatial overlap with anatomical regions of interest (ROIs) spanning ventral to dorsal regions of the PFC (OFC, ACC, dlPFC) and subcortical reward and limbic regions (striatum, amygdala).

Recent evidence suggests ADHD-related sex differences across behavioral and neural domains are another important inter-individual variable to consider. There is a surprising lack of research comparing girls and boys with ADHD to same-sex TD children despite reports that the proportion of males to females diagnosed with the disorder has fallen to approximately 2:1 [36]. Evidence suggests boys with ADHD display greater motor deficits both in terms of behavior [37–39] and the associated neural circuitry [40–44]. In contrast, girls with ADHD tend to display equivalent or greater executive dysfunction both in terms of behavior [39, 45] and the associated neural circuitry [40, 41]. Moreover, girls with ADHD show greater delay discounting relative to TD girls and to boys with ADHD [46], as well as distinct neuropsychological correlates of delay discounting [47] and atypical behavioral response to reward [48]. However, no study has explicitly examined whether FC of fronto-subcortical circuitry is differentially altered among girls and boys with ADHD compared to same-sex TD children.

The current study adds to the existing literature and builds off of our previous findings of greater delay discounting in girls, but not boys, with ADHD by examining ADHD-related sex differences in intrinsic FC of fronto-subcortical brain networks implicated in ADHD and delay discounting. We hypothesized that fronto-subcortical intrinsic FC would be disrupted in ADHD with the greatest differences involving ventromedial regions of the PFC. Given previous evidence of ADHD-related differences in delay discounting being greater among girls, we expected greater disruptions in fronto-subcortical FC among girls. We also examined correlations between delay discounting and intrinsic FC of fronto-subcortical networks.

## Method

### Participants

A total of 147 8–12-year-old children participated in this study: 72 with ADHD (20 girls) and 75 TD children (21 girls).^1^ Demographic information is provided in Table 1, along with inferential statistics regarding diagnostic group differences and sex differences within the ADHD sample. Participants were recruited through local schools, community-wide advertisement, volunteer organizations, medical institutions, and word of mouth. This study was approved by the Johns Hopkins University School of Medicine Institutional Review Board. After providing a complete study description to the participants, oral informed consent was obtained from a parent/guardian prior to the initial phone screening; written informed consent and assent were obtained from the parent/guardian and the child upon arrival at the initial laboratory visit.

**Table 1:** Demographic and clinical characteristics of attention-deficit hyperactivity disorder (ADHD) and typically developing (TD) control groups overall and within sex

	TD						ADHD						Group comparisons			
	Girls (*n* = 21)		Boys (*n* = 54)		All (*n* = 75)		Girls (*n* = 20)		Boys (*n* = 52)		All (*n* = 72)		Girls TD vs. ADHD	Boys TD vs. ADHD	All TD vs. ADHD	ADHD boys vs. girls
	Mean	SD	Mean	SD	Mean	SD	Mean	SD	Mean	SD	Mean	SD	*p* values			
Age (years)	10.3	1.1	10.3	1.2	10.3	1.2	9.8	1.1	10.2	1.4	10.1	1.3	.166	.575	.250	.319
% Male	n/a		n/a		72%		n/a		n/a		72%		n/a	n/a	.976	n/a
% Minority	38%		48%		45%		35%		33%		33%		.837	.105	.137	.852
SES	53.9	9.4	52.8	10.0	53.1	9.8	54.1	9.4	52.8	9.9	53.1	9.7	.959	.976	.966	.638
Handedness	0.71	0.44	0.61	0.57	0.64	0.54	0.64	0.51	0.60	0.58	0.61	0.56	.684	.906	.774	.771
WISC^a^ FSIQ	112.4	11.6	116.5	13.0	115.4	12.7	111.1	10.6	108.0	11.6	108.9	11.4	.704	.001	.001	.314
WISC GAI	112.8	13.0	118.6	13.9	117.0	13.8	113.6	11.2	112.0	13.7	112.4	13.0	.846	.017	.044	.651
CPRS IA T	47.2	6.1	44.1	5.7	45.0	6.0	82.6	9.1	71.0	8.4	74.3	10.0	< .001	< .001	< .001	< .001
CPRS HI T	48.5	7.5	46.3	5.5	46.9	6.2	75.6	16.3	68.9	14.4	70.8	15.2	< .001	< .001	< .001	.096
ADHD-RS IA	3.2	2.8	3.4	3.0	3.3	2.9	20.2	4.9	18.3	4.4	18.8	4.6	< .001	< .001	< .001	.123
ADHD-RS HI	2.9	2.8	2.4	2.6	2.5	2.6	14.4	8.5	12.4	6.6	12.9	7.2	< .001	< .001	< .001	.298
ADHD Presentation CO:IA (count)	n/a		n/a		n/a		16:4		38:14		54:18		n/a	n/a	n/a	.543
% Stim Med	0		0		0		59%		60%		57%		n/a	n/a	n/a	.460
% ODD	0		0		0		50%		31%		36%		n/a	n/a	n/a	.128

*% Minority* percentage of subjects with a self-reported race of African American, Asian, Hispanic, or Biracial, *SES* Hollingshead Four-Factor Index of Socioeconomic Status, *WISC* Wechsler Intelligence Scale for Children, *FSIQ* Full-scale IQ, *GAI* General Ability Index, *CPRS IA T* Conners Parent Rating Scales DSM Inattention Scale *T*-score, *CPRS HI T* Conners Parent Rating Scales DSM Hyperactivity/Impulsivity Scale *T*-score, *ADHD-RS HI* ADHD Rating Scale Hyperactivity/Impulsivity raw score, *ADHD-RS IA* ADHD Rating Scale Inattention raw score, *CO* combined presentation, *IA* predominantly inattentive presentation, *% Stim Med* percentage of subjects taking stimulant medication at the time of the study (all subjects discontinued medication the day prior to and day of study participation), *% ODD* percentage of subjects diagnosed with comorbid Oppositional Defiant Disorder

^a^121 participants were administered the WISC-IV, 26 participants were administered the WISC-V

An initial telephone screening with a parent was conducted. Children with a history of intellectual disability, learning disability, seizures, traumatic brain injury, or other neurological illnesses were excluded. Eligible participants and their parents attended two laboratory sessions. Intellectual ability was assessed during the initial visit using the Wechsler Intelligence Scale for Children, Fourth Edition (*n* = 121, WISC-IV [49]) or Fifth Edition (*n* = 26, WISC-V [50]) and participants with full-scale intelligence quotient (FSIQ) scores below 80 were excluded. To screen for reading disorders, children were administered the Word Reading subtest from the Wechsler Individual Achievement Test, Second Edition (WIAT-II [51]) and were excluded for standard scores below 85.

Diagnostic status was established through administration of either the Diagnostic Interview for Children and Adolescents, Fourth Edition (*n* = 113, DICA-IV [52]) or the Kiddie Schedule for Affective Disorders and Schizophrenia for School Aged Children Present Lifetime version (*n* = 34, KSADS-PL [53]). Children meeting criteria for diagnosis of conduct, mood, generalized anxiety, separation anxiety or obsessive–compulsive disorders on either interview were excluded. A comorbid diagnosis of oppositional defiant disorder (ODD) was permitted for children in the ADHD group given the high base-rate comorbidity between ADHD and ODD. Parents and teachers (when available) also completed the Conners Parent and Teacher Rating Scales-Revised Long Version or the Conners-3 (CPRS and CTRS; [54, 55] and the ADHD Rating Scale-IV, home and school versions (ADHD-RS; [56]). A diagnosis of ADHD was confirmed by a child neurologist or psychologist based on the diagnostic interview, which considered information provided by the parent about functioning at school, in addition to onset, course, duration, and frequency of symptoms, and parent/teacher rating scales (i.e., *T*-scores ≥ 65 or ≥ 6 symptoms endorsed on at least one rating scale). Inclusion in the TD group required scores below clinical cutoffs (i.e., *T*-scores ≤ 60 and ≤ 4 symptoms endorsed on all parent/teacher rating scales. Children taking psychotropic medications other than stimulants were excluded from participation, and children taking stimulants were asked to withhold medication the day prior to and day of testing.

### Procedures

#### Resting state fMRI methods

All children completed a mock scan to acclimate to the scanning environment. rs-fMRI was acquired during a 6-min 30-s scan on a 3.0 T Philips scanner using a single-shot, partially parallel, gradient-recalled echo planar sequence with sensitivity encoding and an ascending slice order (repetition time [TR]/echo time [TE] = 2500/30 ms, flip angle = 75°, sensitivity encoding acceleration factor of 2, 47 3-mm axial slices with no slice gap, in-plane resolution of 3.05 × 3.15 mm [84 × 81 voxels]). Participants were instructed to relax, fixate on a cross-hair, and remain as still as possible.

##### Preprocessing of fMRI data

Functional data were preprocessed using SPM12 (Wellcome Trust Centre for Neuroimaging, London, United Kingdom) and custom MATLAB (The Mathworks, Inc., Natick, Massachusetts) code. rs-fMRI scans were slice-time adjusted using the slice acquired in the middle of the TR as a reference, and rigid body realignment parameters were estimated to adjust for motion. The volume collected in the middle of the scan was spatially normalized using the Montreal Neurological Institute (MNI) EPI template [57]. The estimated rigid body and nonlinear spatial transformations were applied to the functional data together, producing 2-mm isotropic voxels in MNI space. Linear trends were removed, the data were spatially smoothed using a Gaussian filter (6-mm full width at half maximum kernel), and voxel time series were variance normalized. Participants were excluded for between-volume translational movements > 3-mm or rotational movements > 3°. Mean framewise displacement (FD) was calculated using the realignment estimates [58].

##### ICA with backward reconstruction

To examine intrinsic FC between fronto-subcortical regions, we decomposed the data into temporally coherent networks using the Group ICA of fMRI Toolbox (GIFT:  http://mialab.mrn.org/software/gift/index.html; Medical Image Analysis Lab, Albuquerque, New Mexico) [59, 60]. We chose ICA rather than seed-based approaches because of its effectiveness at separating signal from noise [61], its increased sensitivity to detecting individual differences [62], and its ability to identify resting state networks without defining a seed region by grouping voxels with similar time courses. We used an information-theoretic approach to dimension estimation [63] and chose the number of independent components (ICs) for the group to be the maximum dimension estimate across participants, 66. Prior to ICA, each participant’s preprocessed data were reduced to 132 temporally orthogonal principle components (PCs) using principal component analysis (PCA), which explained at least 95% of the variance. Participant-specific PCs were temporally concatenated and a second PCA was used to reduce the aggregate data set to the maximum dimension estimated, 66 (defined above). ICA was repeated on the group-level PCs 10 times using the Infomax algorithm [64] and the ICASSO toolbox [65] with randomized initial conditions in GIFT to ensure stable ICs. Participant-specific spatial maps (SMs) and time courses (TCs) were generated from the aggregate IC decomposition using a method based on PCA compression and projection [59]. The SMs represent the spatial topography of each component within the brain while the TCs represent the intrinsic level of engagement of each component over time.

##### Network identification

We used available brain atlases to extract our cortical and subcortical components of interest from the 66 estimated sources. The Wake Forest Pick Atlas [66] was used to generate anatomical templates for subcortical regions of interest (i.e., striatum and amygdala ROIs). A frontal lobe atlas developed in our lab [67] was used for frontal ROIs (dlPFC, ACC, and OFC). We sorted components based on how well these templates predicted their SMs and selected components with the highest spatial similarity to the template ROIs for further analysis (3D image of components provided in Additional file 1). The frontal ROIs were captured by three components spanning ventral (F1, overlaps with OFC), medial/ACC (F2, overlaps with ACC), and anterior dorsolateral (F3, overlaps with dlPFC) regions of the PFC. The subcortical ROIs were captured by two components including the striatum (S1) and the amygdala and hippocampus (S2). Further details about the regions included in each component are provided in the (Additional file 2: Table S1) and 3D images showing overlap of components with anatomical ROIs are provided in Additional files 3, 4, 5, 6 and 7.

We estimated fronto-subcortical synchrony using Pearson’s correlation coefficient between relevant pairs of participant-specific TCs [68, 69]. Before correlation, outliers were detected from participant-specific TCs and replaced with values from a third order spline fit of clean portions of neighboring data using 3dDespike (Analysis of Functional Neuroimages: http://afni.nimh.nih.gov/afni; NIMH Scientific and Statistical Computing Core, Bethesda, Maryland); this despiking removes lingering noise artifacts not decomposed well by ICA [70]. Pairwise correlations were converted to *Z*-scores using Fisher’s transformation. FC scores further from zero reflect stronger FC regardless of sign; positive scores reflect positive correlations, or in-sync and more integrated activity, while negative scores reflect negative correlations or out-of-sync and more segregated activity.

##### Delay discounting measures

Participants completed a computer-based classic money delay discounting task involving 91 choices between a varying amount of money now ($0–$10.50 in $0.50 increments) or $10.00 after a varying delay (1, 7, 30, or 90 days) [46, 71, 72] and a real-time delay discounting task involving nine choices between playing a preferred game for a shorter amount of time (15, 30, or 45 s) either immediately or for a fixed longer amount of time (60 s) after waiting (either 25, 50, or 100 s) [46, 47]. As in prior studies [46, 71], an indifference point was identified for each delay in order to calculate area under the curve (AUC; [73]) in excel [74] which we then converted to area over the curve (AOC = 1 − AUC) such that higher values indicate greater delay discounting. Task details are provided in previous publications [46, 47].

### Data analysis

Data analysis was accomplished using SPSS Statistics Version 24 (IBM, Chicago). To examine diagnostic group differences in between-network FC between frontal (F1, F2, F3) and subcortical (S1, S2) components, we conducted a 2 diagnosis (ADHD vs. TD) × 2 sex analysis of variance (ANOVA) for each fronto-subcortical pair. Of note, head motion (mean FD) was correlated with FC for some, but not all, of the fronto-subcortical pairs (Additional file 2: Table S2). Due to evidence that head motion contains meaningful information for the study of ADHD [75] and accounting for head motion would underestimate the effect of interest [76], we included mean FD as a covariate in secondary analyses only. In our sample, diagnostic groups did not significantly differ in mean FD (see Additional file 2: Table S3), although mean FD was correlated with ADHD symptoms (see Additional file 2: Table S4), suggesting that head motion during the scan may be part of the ADHD phenotype and including mean FD as a covariate in our main analyses may account for variance attributable to ADHD. Results with mean FD and age as covariates in secondary analyses are provided in Additional file 2: Table S5. Further, we also included FC between the S1-S2 (striatum-amygdala) components as a covariate in secondary analyses to examine whether subcortical-subcortical FC contributed to fronto-subcortical FC (see Additional file 2: Table S6). The general pattern of results remained the same when including these covariates.

Further, girls with ADHD had higher *T*-scores on the CPRS Inattention Scale (*p* < .001; see Table 1). Therefore, diagnostic effects for FC measures were examined among a subset of boys with ADHD with the greatest inattention symptom severity (*n* = 17), thereby eliminating the difference in inattention symptom severity observed among the full sample of boys with ADHD compared to girls with ADHD (*p* = .276). We also compared FC among high- and low-symptom severity groups rather than comparing girls and boys. Collectively, these analyses suggest that inattention symptom severity is not driving the observed sex differences (see Additional file 2: Table S7).

To examine diagnostic group differences in delay discounting, we conducted a 2 diagnosis (ADHD vs. TD) × 2 sex ANCOVA with general ability index (GAI)^2^ as a covariate for each discounting task. We also examined diagnostic group differences separately among girls and boys given our a priori hypotheses of ADHD-related sex differences based on prior work [46]. Next, partial correlations were examined between the six fronto-subcortical pairs and performance on each delay discounting task with GAI and mean FD as covariates. A false discovery rate (FDR) correction of .05 [77] was applied to each family of tests (i.e., correcting for six comparisons for the fronto-subcortical pairs in the diagnostic effects model and 12 comparisons in the brain–behavior correlations) and results surviving this correction are noted. Cohen’s *d* is reported as a measure of effect size (small ~ 0.2, medium ~ 0.5, and large ~ 0.8) [78] consistent with recent recommendations for improving the reliability and interpretability of fMRI research [79].

## Results

### Diagnostic group differences in within network functional connectivity

**Fig. 1:**
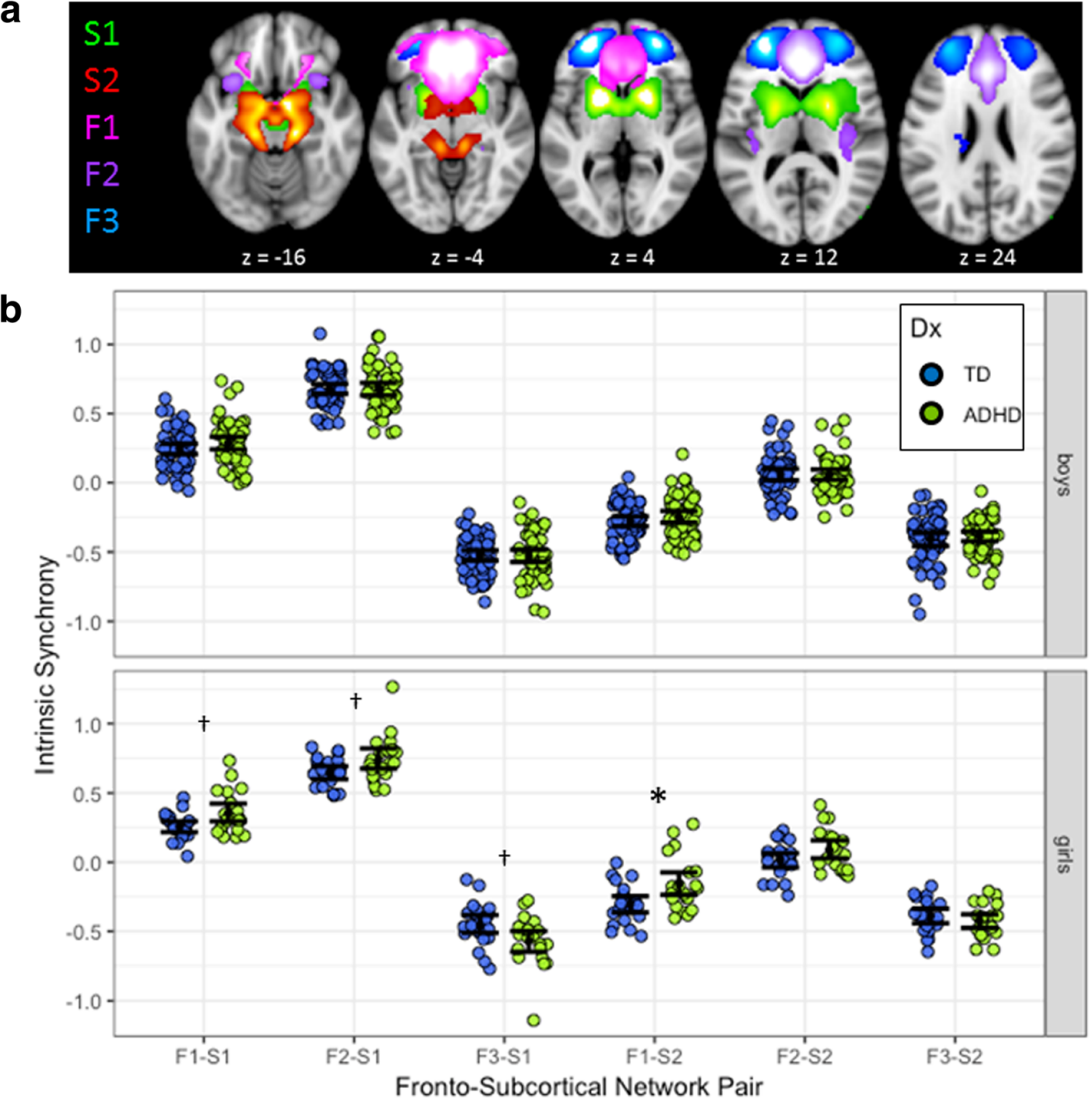
Intrinsic fronto-subcortical FC in girls and boys with attention-deficit/hyperactivity disorder (ADHD) and typically developing (TD) controls. **a** Topography of fronto-subcortical networks estimated from the functional magnetic resonance imaging data using group-independent component analysis. Components with the strongest spatial correlation with anatomical fronto-subcortical regions of interest (ROIs) are shown. Frontal components include F1 (ventromedial PFC; pink), F2 (anterior cingulate cortex; purple), and F3 (anterior dorsolateral prefrontal cortex; blue). Subcortical components include S1 (striatum; green) and S2 (amygdala/hippocampus; red). **b** Dot plots and 95% confidence intervals of the intrinsic synchronization of each pair of the participant-specific fronto-subcortical networks for each diagnostic group separately for boys (top) and girls (bottom). Typically developing (TD, *n* = 75) children are in blue; children with attention-deficit hyperactivity disorder (ADHD, *n* = 72) are in green. Synchronization was calculated as the Pearson correlation between component time courses and converted to a *Z*-score using Fisher’s transform. Confidence intervals are based on comparing the mean of each group to 0. Significant diagnostic group differences within sex were observed among girls only (Table 2) in FC of the S1 (striatum) component with all of the prefrontal components and F1-S2 (vmPFC-amygdala components) FC. *Significant effect after FDR correction applied for six tests; ^†^significant effect without FDR correction

The cortical and subcortical networks are illustrated in Fig. 1a. Before calculating fronto-subcortical synchrony, we compared component topography across groups. Participant-specific SMs of the five components of interest were converted to *z*-values so image intensities reflected the degree to which the component was present in each participant’s data. These SMs were combined in a second-level random effects analysis using a two-sample *t* test in SPM12. Voxels that contributed unequally to the components across groups were identified using a voxelwise *p* = .001 uncorrected and a cluster-level *p =* .05 corrected for multiple comparisons. We found no significant group differences in the spatial topography of any of the cortical or subcortical components representing our ROIs.

### Diagnostic group differences in fronto-subcortical functional connectivity

Analyses of between network FC indicated significant effects of diagnosis and diagnosis × sex interactions as shown in Table 2. Children with ADHD showed atypical FC of F1 (vmPFC) with both subcortical components, such that positive FC with S1 (striatum) was greater in ADHD and negative FC with S2 (amygdala/hippocampus) was weaker in ADHD. In addition, children with ADHD showed greater negative FC between F3 (anterior dlPFC) and S1 (striatum) (FDR uncorrected only). Further, there was some evidence of diagnosis × sex interactions for FC of both F3-S1 (anterior dlPFC-striatum components, *p* = .048) and F1-S2 (anterior dlPFC-amygdala components, *p* = .042), due to much larger effects in girls (*d*s = .74 and .94 in girls compared to .01 and .20 in boys), although these interactions did not survive the FDR correction. Given our a priori hypotheses of sex differences in the diagnostic effects, we tested whether fronto-subcortical FC differed between diagnostic groups separately for girls and boys. Examination of post hoc comparisons for girls and boys separately indicated that diagnostic group differences were driven by girls, with greater FC of S1 (striatum component) with all frontal components and weaker F1-S2 (vmPFC-amygdala components; see Fig. 1), whereas no significant diagnostic effects were observed among boys.

**Table 2:** Intrinsic functional connectivity of fronto-subcortical pairs for children with attention-deficit hyperactivity disorder (ADHD) and typically developing (TD) controls

	TD						ADHD						Group differences				
	Girls (*n* = 21)		Boys (*n* = 54)		All (*n* = 75)		Girls (*n* = 20)		Boys (*n* = 52)		All (*n* = 72)		Dx	Dx × Sex	All	Girls	Boys
	Mean	SD	Mean	SD	Mean	SD	Mean	SD	Mean	SD	Mean	SD	*p*	*p*	*d*	*d*	*d*
F1-S1	0.26	0.15	0.25	0.15	0.25	0.16	0.36	0.15	0.29	0.15	0.32	0.17	.012^a^	.291	0.42^a^	0.66^b^	0.27
F2-S1	0.65	0.15	0.68	0.15	0.66	0.16	0.74	0.15	0.68	0.14	0.71	0.16	.092	.079	0.28	0.64^b^	0.01
F3-S1	− 0.45	0.16	− 0.52	0.16	− 0.49	0.18	− 0.57	0.16	− 0.53	0.16	− 0.55	0.18	.041^b^	.048^b^	0.33^b^	0.74^b^	0.01
F1-S2	− 0.31	0.16	− 0.28	0.15	− 0.29	0.17	− 0.16	0.15	− 0.25	0.15	− 0.20	0.17	.002^a^	.042^b^	0.51^a^	0.94^a^	0.20
F2-S2	0.02	0.15	0.06	0.15	0.04	0.16	0.09	0.15	0.06	0.14	0.07	0.16	.183	.172	0.22	0.49	0.01
F3-S2	− 0.39	0.15	− 0.41	0.15	− 0.40	0.16	− 0.42	0.15	− 0.39	0.15	− 0.41	0.16	.805	.310	0.04	0.23	0.14

Estimated marginal mean (SD) functional connectivity values for the ADHD and TD groups. *F1* frontal component 1 (ventromedial PFC), *F2* frontal component 2 (medial PFC/anterior cingulate cortex), *F3* frontal component 3 (anterior dorsolateral PFC), *S1* subcortical component 1 (striatum), *S2* subcortical component 2 (amygdala/hippocampus). Cohen’s *d* is reported as an estimate of effect size. Uncorrected *p* values are reported. ^a^Significant effect after FDR correction applied for six tests; ^b^Significant effect without FDR correction

### Diagnostic group differences in delay discounting

For the delay discounting analyses, there was a significant diagnosis × sex interaction for real-time discounting, *F*(1,132) = 4.0, *p* = .048. Consistent with previous research [46, 47], girls with ADHD showed greater delay discounting than TD girls on the real-time task (*p* = .028, *d* = 0.68) whereas boys with ADHD did not differ from TD boys (*p* = .791, *d* = 0.02). In contrast, diagnostic groups did not differ on the money-discounting task, diagnosis: *F*(1, 142) = 0.06, *p* = .811 and diagnosis × sex: *F*(1,142) = 0.02, *p* = .897.

### Delay discounting correlations with between network FC

**Fig. 2:**
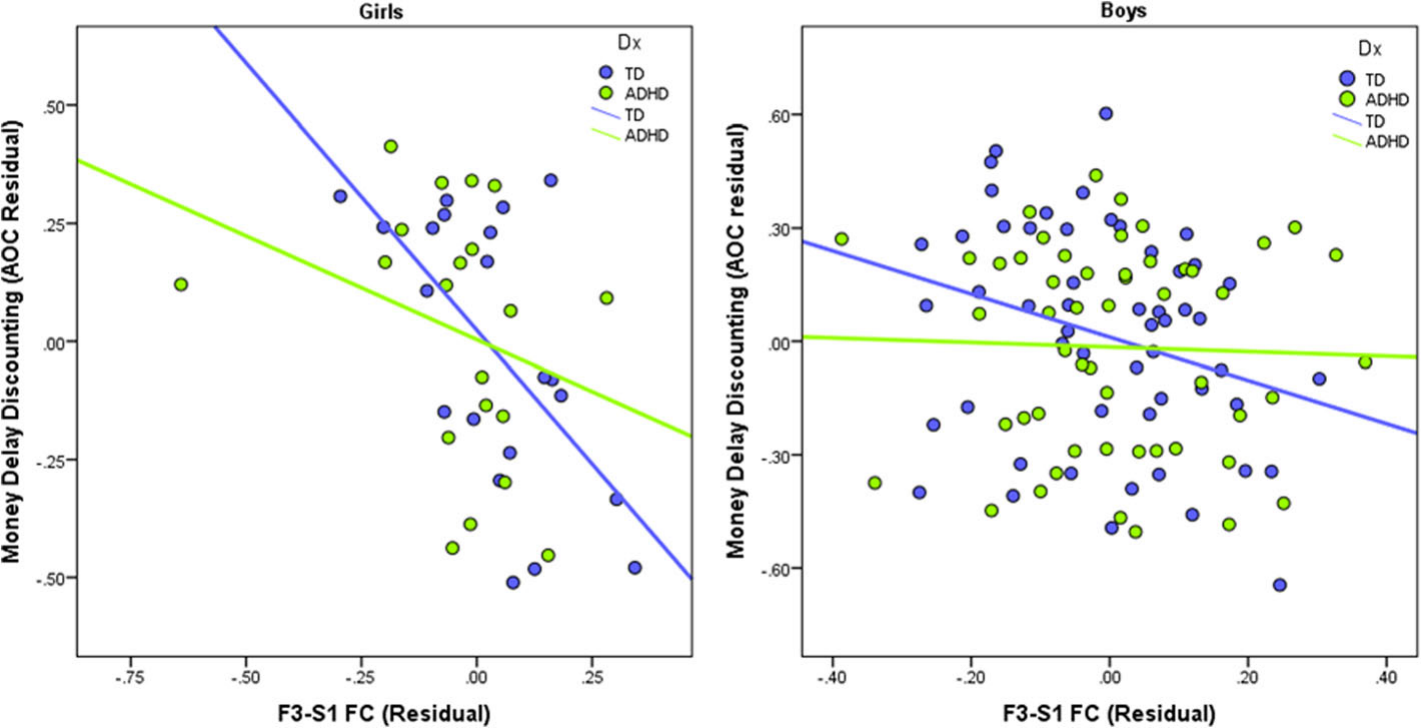
Scatterplot of the partial correlation between intrinsic fronto-subcortical FC and delay discounting. Across groups, children who displayed greater negative F3-S1 (anterior dlPFC-striatum components) FC showed greater monetary delay discounting (*p* = .004)

Examination of partial correlations (with GAI and mean FD as covariates) between the delay discounting and FC measures across all children suggested a significant relationship between F3-S1 (anterior dlPFC-striatum components) FC and performance on the money discounting task, *r*(143) = − .235, *p* = .004 (see Additional file 2: Table S8). However, no significant correlations were observed between real-time discounting and any FC measures in the full sample, *r*s(133) < .14, *p*s > .10. Thus, within the full sample, children who displayed more negative F3-S1 FC also showed greater money delay discounting. To further explore this relationship, we tested whether diagnosis, sex, and their interaction moderate the relationship between F3-S1 FC and money discounting observed in the full sample. In this model, F3-S1 FC, diagnosis, sex, and the 2- and 3-way interactions among variables were entered as predictors of money discounting along with GAI and mean FD as covariates. The results suggest that F3-S1 significantly predicts money discounting (*β* = − .99, *p* = .016), whereas there was no evidence that diagnosis (*β* = .2791, *p* = .326) or a diagnosis × sex interaction (*β* = − .24, *p* = .716) moderates this relationship. As shown in Fig. 2, this relationship was strongest among TD girls (*r*(21) = − .591) and TD boys (*r*(54) = − .292), followed by ADHD girls (*r*(20) = − .244), with no evidence of a relationship among ADHD boys (*r*(52) = − .031).

**Fig. 3:**
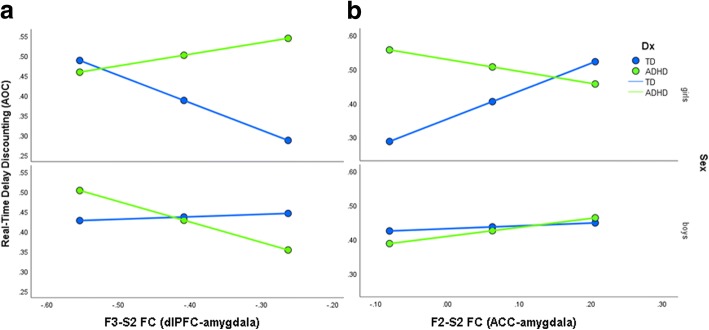
Plot of the regression results showing the conditional effects of F3-S2 (dlPFC-amygdala) FC (left) and F2-S2 (ACC-amygdala) FC (right) in relation to real-time delay discounting for each diagnosis by sex subgroup

Due to the diagnosis × sex interaction for real-time discounting, we tested whether diagnosis and sex moderate the brain–behavior relationship between fronto-subcortical FC (for each of the six pairs) and real-time discounting and applied an FDR correction for six tests (i.e., the diagnosis × sex interaction for each FC pair). The results suggest a diagnosis × sex interaction moderates the relationship between real-time discounting and FC between the F3-S2 (dlPFC- amygdala) components (*β* = − 1.56, *p* < .0001) and the F2-S2 (ACC- amygdala) components (*β* = 1.34, *p* = .002; Table 3). As shown in the plot of the conditional effects (Fig. 3), stronger negative F3-S2 (dlPFC-amygdala) FC was related to heightened real-time discounting among TD girls (*p* = .011) and ADHD boys (*p* = .004) but not among ADHD girls (*p* = .293) or TD boys (*p* = .604). Further, stronger positive F2-S2 (ACC-amygdala) FC was related to greater real-time discounting among TD girls only (*p* = .007), but not among ADHD girls (*p* = .541), TD boys (*p* = .124), or ADHD boys (*p* = .139). There were no significant diagnosis × sex × FC interactions for the remaining fronto-subcortical pairs.

**Table 3:** Results for significant diagnosis × sex moderation of fronto-subcortical FC and real-time delay discounting

		Real-time delay discounting		
		*b*	*t*	*p*
F2-S2 FC	Mean FD	− .11	− 1.72	.088
	GAI	− .01	− .1.63	.105
	S2-F2 FC	.82	2.73	.007
	Dx	.17	3.38	.001
	Sex	.08	1.85	.067
	S2-F2 FC × Dx	− 1.16	− 3.12	.002
	S2-F2 FC × Sex	− .73	− 2.24	.027
	Dx × Sex	− .20	− 3.16	.002
	S2-F2 FC × Dx × Sex	1.34	3.14	.002
	*R* ^2^	.15		
	*F*	2.5		
F3-S2 FC	mean FD	− .09	− 1.51	.132
	GAI	− .01	− 1.87	.064
	S2-F3 FC	− .69	− 2.58	.011
	Dx	.52	3.15	.002
	Sex	.36	2.91	.004
	S2-F3 FC × Dx	.98	2.55	.012
	S2-F3 FC × Sex	.75	2.57	.011
	Dx × Sex	− .76	− 4.07	.0001
	S2-F3 FC × Dx × Sex	− 1.56	− 3.57	.0005
	*R* ^2^	.18		
	*F*	3.0		

*S2-F2 FC* functional connectivity (FC) of the ACC-amygdala components, *F3-S2 FC* FC of the dlPFC-amygdala components, *FD* framewise displacement, *Dx* diagnostic group (ADHD, TD)

## Discussion

The current study adds to the existing ADHD neuroimaging and delay discounting literature by combining a data-driven approach to identify intrinsic functional networks with a theory-driven approach to examine ADHD-related sex differences in fronto-subcortical FC. Our findings suggest that children with ADHD show atypical FC between the vmPFC component and subcortical regions, including stronger positive FC with the striatum component and weaker negative FC with the amygdala component, with greater magnitude of effects among girls although the small effects among boys were in the same direction. In addition, girls with ADHD show atypical intrinsic FC between the striatum component and the relatively dorsal PFC components, including stronger positive FC with the ACC component and stronger negative FC with the dlPFC component. Further, girls but not boys, with ADHD, show heightened delay discounting on the real-time task compared to TD girls, as previously reported [46], whereas no diagnostic effects were observed among boys. Examination of brain–behavior correlations showed that FC between the anterior dlPFC-striatal components correlated with money delay discounting across all participants, regardless of diagnosis. Further, FC of the amygdala component with both the ACC and dlPFC components was differentially related to real-time delay discounting among girls and boys with and without ADHD. These findings contribute to the growing literature examining functional connectivity of fronto-striatal networks implicated in ADHD using ICA methods and extend this literature through examination of ADHD-related sex differences and associations with multiple measures of delay discounting.

Consideration of these finding with the existing literature provides growing evidence for stronger vmPFC-striatum FC, thought to reflect greater integration [80, 81], among children and adolescents with ADHD [30–32]. Fewer studies have examined connectivity of the amygdala among children with ADHD, with evidence of greater PFC-amygdala FC in adolescents with ADHD during an emotional task [82] and in relation to emotional lability [83], whereas reduced negative FC of an amygdala subregion with the dlPFC has been reported among boys with ADHD [84]. Our findings add to this literature, suggesting reduced negative FC, thought to reflect reduced segregation, [80, 81] between the vmPFC-amygdala components in ADHD. Our findings of atypical intrinsic vmPFC-subcortical FC in children with ADHD may be related to the behavioral and emotional dysregulation observed in individuals with ADHD given the role of the vmPFC in top-down inhibitory control of bottom-up activity in subcortical areas. The vmPFC is a key component of the brain’s reward system and is highly interconnected with subcortical structures involved in reward and affective processing such as the striatum and amygdala [85]. Research has shown that the vmPFC regulates behavior by inhibiting the influence of emotions, thoughts, and actions [86]. Further, the vmPFC is involved in representing the actual and expected reward-value of stimuli, reward prediction errors, and reward-based decision-making [87]. Although diagnostic groups did not differ in the spatial topography of the vmPFC component, FC between this component and subcortical components was atypical among children with ADHD, particularly girls, highlighting the importance of examining interactions between fronto-subcortical neural networks. Furthermore, these findings call attention to the influence of sex on ADHD-related differences in fronto-subcortical functional networks and emphasize the importance for replication of these results among larger samples of girls with ADHD using ICA- and seed-based methods.

Examination of fronto-subcortical FC within sex suggests girls with ADHD, but not boys, displayed stronger negative anterior dlPFC-striatum FC compared to same-sex TD children (*d* = .74), and this correlated with money delay discounting. Thus, individuals showing stronger functional segregation between striatal regions involved in reward processing and prefrontal regions involved in cognitive control tend to show greater delay discounting (Fig. 2). In contrast, FC of the amygdala with relatively dorsal PFC components correlated with real-time discounting among TD girls and, to a lesser extent, among ADHD boys. The differential associations between dlPFC-striatum FC and money delay discounting and between dlPFC/ACC-amygdala FC and real-time delay discounting suggests the neural correlates of delay discounting depend on characteristics of the task. In particular, when delays and rewards are experienced in real-time, negative affect associated with waiting may contribute to the preference for immediate reward as suggested by delay aversion models of ADHD [1, 22, 88]. This may be why functional connectivity of the amygdala is more strongly related to real-time delay discounting whereas decision-making on delay discounting tasks involving more abstract reasoning without a significant affective component relate to connectivity between brain regions governing cognitive control and reward.

One previous study using the identical money delay discounting task along with a seed-based analysis reported that increased positive NAcc-anterior PFC FC (a small region included in the anterior dlPFC component examined here) was positively correlated with delay discounting [31]. Although both studies implicate atypical striatal-PFC FC in delay discounting, the direction of these effects differs. In the current study, we used ICA to functionally define a component that includes the caudate and putamen rather than focusing specifically on the NAcc, which may contribute to the discrepant findings. In addition, the dlPFC component is much larger than the anterior PFC component in the previous study, suggesting that distinct functional connectivity patterns may be observed across different regions of the PFC. However, the consistent involvement of striatal-PFC regions in relation to delay discounting suggests a possible neural mechanism of heightened delay discounting in ADHD. Importantly, children with ADHD did not significantly differ in their performance on the money delay discounting task involving choices about money (although they did differ in the task involving choices about gametime), consistent with some prior research [23, 46, 89–91]. This might suggest a subgroup of children with ADHD who display atypical delay discounting and fronto-striatal FC, which may inform our understanding of heterogeneity in ADHD (e.g., [32]).

The novel findings of ADHD-related sex differences in fronto-subcortical FC and associations with delay discounting must be considered within the limitations of this study. First, the majority of sample of children with ADHD included in this study were not naïve to stimulant medication and it is unclear what, if any, affect this might have on our findings. Second, in order to understand the pathophysiology of ADHD specifically, we excluded children with comorbid disorders other than ODD, which limits the generalizability of our results. Our results also may not generalize to children with more severe ADHD and behavioral problems due to the exclusion of participants with excessive motion during the resting-state scan. Future research must attempt to replicate these findings given the small sample of girls with ADHD as well as the inconsistent results in the ADHD neuroimaging literature and the lack of studies comparing girls and boys with ADHD, and to extend these findings using longitudinal methods to understand the developmental trajectory of anomalous fronto-subcortical FC in ADHD.

## Conclusions

Our findings suggest functional fronto-subcortical networks are affected in children with ADHD, particularly girls, such that the striatum is intrinsically more strongly connected to frontal regions, being both more functionally segregated (e.g., negatively correlated) with the anterior dlPFC and more functionally integrated (e.g., positively correlated) with the vmPFC, while the amygdala/hippocampus is intrinsically less connected to the vmPFC. In addition, intrinsic FC of the striatum and amygdala is differentially related to money and real-time discounting, providing support for unique neural correlates of delay discounting tasks involving real versus hypothetical delays and rewards. These findings add to the extant literature implicating fronto-striatal circuitry in children with ADHD and expand upon these findings to reveal associations with a behavioral preference for immediate reward and atypical functional connectivity of the amygdala in ADHD. Moreover, this is the first study to show greater anomalies in fronto-subcortical functional networks among girls with ADHD. This study adds to our understanding of the neurobiological correlates of ADHD and suggests potential differences among school-age girls and boys with ADHD that relate to reward-based decision-making.

## Additional files

Additional file 1: 3D image of the frontal and subcortical functional components. An interactive tool to view the five frontal and subcortical functional components used in the analyses. The frontal components include F1 (vmPFC; pink), F2 (medial PFC/ACC; purple), and F3 (anterior dlPFC; blue). The subcortical components include S1 (striatum; green) and S2. (HTML 27309 kb)

Additional file 2: **Supplementary Material. Table S1.** Anatomical information for resting state components. **Table S2.** Correlation between covariates and dependent variables across ADHD and TD groups. **Table S3.** Framewise displacement (FD) for diagnostic x sex subgroups. **Table S4.** a. correlation between head motion (mean FD) and ADHD symptoms among the full sample and separately among girls and boys; b. Partial correlations between head motion (mean FD) and functional connectivity (FC) accounting for ADHD Inattention *T*-scores. **Table S5.** Effects of diagnosis and interactions with sex for intrinsic functional connectivity (FC) of fronto-subcortical pairs among children with ADHD and TD controls with and without mean FD and age as covariates. **Table S6.** Intrinsic functional connectivity of fronto-subcortical pairs for children with ADHD and TD children with and without S1-S2 FC as a covariate. **Table S7.** Intrinsic functional connectivity of fronto-subcortical pairs for children with ADHD and TD children in the full sample (*n* = 147) and among a reduced sample including a subset of boys with ADHD (*n* = 17/52) with similar inattention symptom severity *T*-scores as the sample of girls with ADHD (*n* = 112). **Table S8.** Correlation between FC of fronto-subcortical network pairs and delay discounting (area over the curve) across ADHD and TD groups. (DOCX 34 kb)

Additional file 3: An interactive tool to view the spatial overlap of the anatomical ROI for the OFC (yellow) and the functional component with the highest spatial overlap (F1; same color as shown in Fig. 1a) used in the analyses. (HTML 2047 kb)

Additional file 4: An interactive tool to view the spatial overlap of the anatomical ROI for the ACC (yellow) and the functional component with the highest spatial overlap (F2). (HTML 2054 kb)

Additional file 5: An interactive tool to view the spatial overlap of the anatomical ROI for the dlPFC (yellow) and the functional component with the highest spatial overlap (F3). (HTML 2047 kb)

Additional file 6: An interactive tool to view the spatial overlap of the anatomical ROI for the striatum and the functional component with the highest spatial overlap (S1). (HTML 3113 kb)

Additional file 7: An interactive tool to view the spatial overlap of the anatomical ROI for the amygdala and the functional component with the highest spatial overlap (S2). (HTML 3209 kb)

## Abbreviations

ACC: Anterior cingulate cortex
AOC: Area over the curve
AUC: Area under the curve
dlPFC: Dorsolateral prefrontal cortex
EF: Executive function
F1: Frontal component 1 (highest spatial overlap with orbitofrontal cortex anatomical region of interest)
F2: Frontal component 2 (highest spatial overlap with anterior cingulate cortex anatomical region of interest)
F3: Frontal component 3 (highest spatial overlap with dorsolateral prefrontal cortex anatomical region of interest)
FC: Functional connectivity
FD: Framewise displacement
FDR: False discovery rate
GAI: General Ability Index
ICA: Independent components analysis
ICs: Independent components
NAcc: Nucleus accumbens
ODD: Oppositional Defiant Disorder
OFC: Orbitofrontal cortex
PCA: Principle component analysis
PCs: Principle components
PFC: Prefrontal cortex
ROI: Region of interest
rs-fMRI: Resting-state functional magnetic resonance imaging
S1: Subcortical component 1 (highest spatial overlap with striatum anatomical region of interest)
S2: Subcortical component 2 (highest spatial overlap with amygdala anatomical region of interest)
SMs: Spatial maps
TCs: Time courses
TD: Typically developing
vmPFC: Ventromedial prefrontal cortex
VS: Ventral striatum

## Footnotes

1 The current study sample includes data from 82 participants (51% of the ADHD sample and 60% of the TD sample) in a previously published study examining behavioral measures of delay discounting [46]. Neuroimaging data was not included in that report.

2 The general ability index (GAI) from the WISC is a measure of intellectual reasoning ability based on verbal and perceptual reasoning abilities while excluding working memory and processing speed performance. As the latter domains are typically affected among children with ADHD, GAI was selected as a covariate rather than FSIQ which includes performance in these domains.
