# Supplementary material for: ADHD-related sex differences in fronto-subcortical intrinsic functional connectivity and associations with delay discounting
Source: J Neurodev Disord. 2018 Dec 13;10:34. doi: 10.1186/s11689-018-9254-9 (PMC6292003; doi:10.1186/s11689-018-9254-9)
Supplement: Supplementary file 1 — 3D image of the frontal and subcortical functional components. An interactive tool to view the five frontal and subcortical functional components used in the analyses. The frontal components include F1 (vmPFC; pink), F2 (medial PFC/ACC; purple), and F3 (anterior dlPFC; blue). The subcortical components include S1 (striatum; green) and S2. (HTML 27309 kb) [file 11689_2018_9254_MOESM1_ESM.html]

Papaya Viewer
