# Supplementary material for: ADHD-related sex differences in fronto-subcortical intrinsic functional connectivity and associations with delay discounting
Source: J Neurodev Disord. 2018 Dec 13;10:34. doi: 10.1186/s11689-018-9254-9 (PMC6292003; doi:10.1186/s11689-018-9254-9)
Supplement: Supplementary file 2 — Supplementary Material. Table S1. Anatomical information for resting state components. Table S2. Correlation between covariates and dependent variables across ADHD and TD groups. Table S3. Framewise displacement (FD) for diagnostic x sex subgroups. Table S4. a. correlation between head motion (mean FD) and ADHD symptoms among the full sample and separately among girls and boys; b. Partial correlations between head motion (mean FD) and functional connectivity (FC) accounting for ADHD Inattention T-scores. Table S5. Effects of diagnosis and interactions with sex for intrinsic functional connectivity (FC) of fronto-subcortical pairs among children with ADHD and TD controls with and without mean FD and age as covariates. Table S6. Intrinsic functional connectivity of fronto-subcortical pairs for children with ADHD and TD children with and without S1-S2 FC as a covariate. Table S7. Intrinsic functional connectivity of fronto-subcortical pairs for children with ADHD and TD children in the full sample (n = 147) and among a reduced sample including a subset of boys with ADHD (n = 17/52) with similar inattention symptom severity T-scores as the sample of girls with ADHD (n = 112). Table S8. Correlation between FC of fronto-subcortical network pairs and delay discounting (area over the curve) across ADHD and TD groups. (DOCX 34 kb) [file 11689_2018_9254_MOESM2_ESM.docx]

**Supplementary Material**

**Table S1. Anatomical information for resting state components**

| Component | Correlation  with ROI | Region Label | Neurosynth | BA | Extent | x | y | z |
| --- | --- | --- | --- | --- | --- | --- | --- | --- |
| F1 | 0.24 | L OFC | OFC | 11 | 5301 | -22 | 34 | -12 |
|  |  | R Dorsal ACC | ACC | 32 |  | 8 | 36 | -4 |
|  |  | L Caudate | NAcc |  |  | -8 | 10 | -8 |
| F2 | 0.43 | L Ventral ACC | ACC | 24 | 4465 | 0 | 32 | 16 |
| F3 | 0.42 | L Anterior PFC | Anterior PFC | 10 | 4007 | -28 | 52 | 8 |
|  |  | L FEF | dlPFC | 8 |  | -44 | 20 | 36 |
|  |  | R Anterior PFC | Anterior PFC | 10 | 3096 | 28 | 54 | 4 |
|  |  | R dlPFC | dlPFC | 9 |  | 44 | 26 | 38 |
| S1 | 0.48 | L Putamen | Putamen |  | 7078 | -20 | 10 | 2 |
|  |  | R Putamen | Putamen |  |  | 18 | 4 | 8 |
|  |  | R Insula | Insula | 45 |  | 36 | 20 | 8 |
| S2 | 0.19 | R Amygdala | Amygdala |  | 4486 | 12 | -2 | -20 |
|  |  | L Amygdala | Amygdala |  |  | -12 | -4 | -22 |
|  |  | L Parahippocampal gyrus | MTL |  |  | -16 | -26 | -14 |
| Note. Talaraich labels for areas included in resting state frontal (F1, F2, F3) and subcortical (S1, S2) components identified using group ICA. Locations reported for peak activation (at least 20 mm apart) in the major cluster of each component only, further information about each component and the associated anatomy is provided in interactive 3D images included in Additional Files 1-6. F1 = frontal component 1 (ventromedial PFC), F2 = frontal component 2 (medial PFC/anterior cingulate cortex), F3 = frontal component 3 (anterior dorsolateral PFC), S1 = subcortical component 1 (striatum), S2 = subcortical component 2 (amygdala/hippocampus). L = Left, R = Right, OFC = orbitorfrontal cortex, ACC = anterior cingulate cortex, NAcc = nucleus accumbens, PFC = prefrontal cortex, FEF = frontal eye field, dlPFC = dorsolateral PFC, and MTL = medial temporal lobe. | | | | | | | | |

**S2. Correlations between potential covariates and dependent variables**

We examined correlations between the primary dependent variables (functional connectivity (FC) of the six fronto-subcortical pairs and area over the curve for each delay discounting task) and age, mean framewise displacement (FD), and intellectual reasoning ability (GAI) in the full sample. As shown in **Table S2**, age did not significantly correlate with any of the dependent variables (*r*s<.12), whereas GAI correlated was moderately correlated with money discounting (*r*=-.305), and mean FD was moderately correlated with FC of four out of six fronto-subcortical pairs.

| **Table S2.** Correlation between covariates and dependent variables across ADHD and TD groups. | | | | | | | | |
| --- | --- | --- | --- | --- | --- | --- | --- | --- |
| (*n*=147) | F1-S1 | F2-S1 | F3-S1 | F1-S2 | F2-S2 | F3-S2 | Money DD | Game DD^a^ |
| Age | .043 | -.057 | .051 | -.119 | .024 | .031 | -.107 | -.089 |
| Mean FD | .104 | .301** | -.219** | .099 | .385** | -.213** | -.039 | -.073 |
| GAI | -.015 | -.057 | .184* | -.024 | .025 | -.138 | -.305** | -.124 |
| Note. FD = framewise displacement; GAI = general ability index; DD = delay discounting  Pearson’s correlation coefficient reported above. **p*<.05, ***p*≤.01. ^a^*n*=137  FC for fronto-subcortical pairs shaded in gray significantly differ between diagnostic groups (see Table S5). | | | | | | | | |

**S3. Comparing head motion across diagnostic groups**

To determine whether diagnostic groups differed in head motion during the scan, we conducted a 2 Diagnosis (ADHD vs. TD) × 2 Sex analysis of variance (ANOVA) for mean FD. Diagnostic groups did not significantly differ in mean FD, *F*(1, 143)=2.9, *p*=.092, nor was there evidence of a Diagnosis × Sex interaction, *F*(1, 143)=2.1, *p*=.155 (see descriptive statistics provided in **Table S3**).

| **Table S3.** Framewise displacement (FD) for diagnostic x sex subgroups. | | | | | | | | | | | |
| --- | --- | --- | --- | --- | --- | --- | --- | --- | --- | --- | --- |
| TD | | | | | | ADHD | | | | | |
| Girls  (n=21) | | Boys  (n=54) | | All  (n=75) | | Girls  (n=20) | | Boys  (n=52) | | All  (n=72) | |
| Mean | SD | Mean | SD | Mean | SD | Mean | SD | Mean | SD | Mean | SD |
| 0.19 | 0.10 | 0.32 | 0.25 | 0.28 | 0.22 | 0.31 | 0.20 | 0.33 | 0.22 | 0.32 | 0.21 |

**S4. Correlations between head motion and ADHD symptoms**

Next, we examined correlations between FD and ADHD symptoms to determine whether FD may be part of the ADHD phenotype, suggesting that we should not account for FD in our main analyses as this may reflect variance attributable to ADHD. As shown in **Table S4a**, FD is significantly correlated with inattention symptoms among girls only. Further, as shown in **Table S4b**, partial correlations between FD and FC for each fronto-subcortical pair accounting for inattention symptoms remained non-significant for F1-S1 and F1-S2 and became non-significant for F3-S1. This information, combined with the results presented in **Table S5**, suggest that for the fronto-subcortical pairs for which we see diagnostic group differences, these results are not driven by motion.

| **Table S4a.** Correlation between head motion (mean FD) and ADHD symptoms among the full sample and separately among girls and boys. | | | |
| --- | --- | --- | --- |
|  |  | Inattention T-score | Hyper/Imp T-score |
| Full Sample (*n*=147) | Mean FD | .096 | .080 |
| Boys (n=106) | Mean FD | .050 | .077 |
| Girls (n=41) | Mean FD | .374* | .182 |

| Table S4b. Partial correlations between head motion (mean FD) and functional connectivity (FC) accounting for ADHD Inattention T-scores | | | | | | | |
| --- | --- | --- | --- | --- | --- | --- | --- |
|  |  | F1-S1 | F2-S1 | F3-S1 | F1-S2 | F2-S2 | F3-S2 |
| Full Sample (*n*=147) | Mean FD | .085 | .294** | -.208 | .077 | .382** | -.211* |
| Boys (n=106) | Mean FD | .102 | .317* | -.189 | .114 | .408** | -.233* |
| Girls (n=41) | Mean FD | .039 | .204 | -.201 | -.084 | .252 | -.117 |

**S5. Impact of head motion and age on main results**

The following analyses were conducted to determine whether head motion during the scan or age impacted the results. We conducted separate 2 Diagnosis (ADHD vs. TD) × 2 Sex analysis of covariance (ANCOVA) with mean FD as a covariate and with age as a covariate for each fronto-subcortical pair. Analyses of between network FC with mean FD as a covariate and with age as a covariate were highly similar to the main results without covariates, although most *p*-values increased (**Table S5**).

| **Table S5.** Effects of diagnosis and interactions with sex for intrinsic functional connectivity (FC) of fronto-subcortical pairs among children with attention-deficit hyperactivity disorder (ADHD) and typically developing (TD) controls with and without mean FD and age as covariates. | | | | | | | | | | | | | | | |
| --- | --- | --- | --- | --- | --- | --- | --- | --- | --- | --- | --- | --- | --- | --- | --- |
|  | No covariates | | | | | Covary Mean FD | | | | | Covary Age | | | | |
|  | Dx | Dx×Sex | All | Girls | Boys | Dx | Dx×Sex | All | Girls | Boys | Dx | Dx×Sex | All | Girls | Boys |
|  | *p* | *p* | *d* | *d* | *d* | *p* | *p* | *d* | *d* | *d* | *p* | *p* | *d* | *d* | *d* |
| F1-S1 | .012* | .291 | 0.42* | 0.66^†^ | 0.27 | .020^†^ | .362 | 0.39^†^ | 0.60 | 0.27 | .010* | .268 | 0.44^*^ | 0.68^†^ | 0.27 |
| F2-S1 | .092 | .079 | 0.28 | 0.64^†^ | 0.01 | .217 | .164 | 0.21 | 0.49 | 0.03 | .105 | .085 | 0.27 | 0.62^†^ | 0.02 |
| F3-S1 | .041^†^ | .048^†^ | 0.33^†^ | 0.74^†^ | 0.01 | .083 | .085 | 0.29 | 0.64^†^ | 0.00 | .048^†^ | .052 | 0.33^†^ | 0.73^†^ | 0.01 |
| F1-S2 | .002* | .042^†^ | 0.51* | 0.94* | 0.20 | .004* | .057 | 0.49* | 0.90* | 0.19 | .004* | .050 | 0.49* | 0.90* | 0.18 |
| F2-S2 | .183 | .172 | 0.22 | 0.49 | 0.01 | .455 | .374 | 0.12 | 0.31 | 0.03 | .171 | .166 | 0.23 | 0.51 | 0.00 |
| F3-S2 | .805 | .310 | 0.04 | 0.23 | 0.14 | .908 | .471 | 0.02 | 0.12 | 0.15 | .835 | .322 | 0.04 | 0.22 | 0.14 |
| Note. Statistical *p*- and *d*-values are based on the estimated marginal means. Cohen’s *d* is reported as an estimate of effect size.  *Significant effect after false discovery rate (FDR) correction applied for six tests; ^†^*p*<.05 without FDR correction. | | | | | | | | | | | | | | | |

**S6. Analysis of FC between the S1-S2 (striatum-amygdala) components**

Although FC between the subcortical components was not the focus of the paper, there was evidence of significant positive S1-S2 (striatum-amygdala/hippocampus) FC (*M*=.13, SD=.13, *t*(146)=11.9, *p*<.0001) that was not impacted by diagnosis (*p*=.435), sex (*p*=.652), or their interaction (*p*=.393). However, S1-S2 FC is significantly correlated with F1-S2 FC (*r*=.364, *p*<.001), but not with F1-S1 FC (*r*=-.131, *p*=.114). To determine whether S1-S2 FC accounts for the effects of diagnosis, sex, or their interaction for any of the fronto-subcortical pairs reported in the main results, we conducted a series of 2 Diagnosis x 2 Sex ANCOVAs with S1-S2 FC as a covariate. The results are similar, with significant main effects of diagnosis for F1-S1 (*p*=.009) and F1-S2 (*p*=.003) and marginal Diagnosis x Sex interactions for F3-S1 (*p*=.073) and F1-S2 (*p*=.066). These analyses suggest that S1-S2 FC is not driving the main results.

| **Table S6.** Intrinsic functional connectivity of fronto-subcortical pairs for children with attention-deficit hyperactivity disorder (ADHD) and typically developing (TD) children with and without S1-S2 FC as a covariate. | | | | | | | | | | |
| --- | --- | --- | --- | --- | --- | --- | --- | --- | --- | --- |
|  | No covariates | | | | | Covary S1-S2 FC | | | | |
|  | Dx | Dx×Sex | All | Girls | Boys | Dx | Dx×Sex | All | Girls | Boys |
|  | *p* | *p* | *d* | *d* | *d* | *p* | *p* | *d* | *d* | *d* |
| F1-S1 | .012* | .291 | 0.42* | 0.66^†^ | 0.27 | .009* | .233 | 0.44 | 0.72^†^ | 0.27 |
| F2-S1 | .092 | .079 | 0.28 | 0.64^†^ | 0.01 | .107 | .093 | 0.27 | 0.60 | 0.01 |
| F3-S1 | .041^†^ | .048^†^ | 0.33^†^ | 0.74^†^ | 0.01 | .054 | .073 | 0.33 | 0.70^†^ | 0.02 |
| F1-S2 | .002* | .042^†^ | 0.51* | 0.94* | 0.20 | .003* | .066 | 0.49 | 0.90* | .021 |
| F2-S2 | .183 | .172 | 0.22 | 0.49 | 0.01 | .279 | .282 | 0.18 | 0.40 | 0.00 |
| F3-S2 | .805 | .310 | 0.04 | 0.23 | 0.14 | .883 | .492 | 0.03 | 0.10 | 0.15 |
| Note. Statistical *p*- and *d*-values are based on the estimated marginal means. Cohen’s *d* is reported as an estimate of effect size.  *Significant effect after FDR correction applied for six tests; ^†^*p*<.05 without FDR correction. | | | | | | | | | | |

**S7. Results with subsample of boys with elevated Conners Inattention T-scores**

***Diagnostic Group Differences in Fronto-Subcortical Functional Connectivity***

To determine whether the diagnostic differences in FC of fronto-subcortical pairs among girls, but not boys, was due to elevated inattention symptom severity (parent-report on the Conners Rating Scale) among girls with ADHD compared to boys with ADHD (*p*<.001, Table 1), we conducted a 2 Diagnosis (ADHD vs. TD) × 2 Sex analysis of variance (ANOVA) for each fronto-subcortical pair including a subsample of boys with ADHD with the greatest inattention T-scores, thereby eliminating the sex difference in inattention T-scores observed in the ADHD group (*p*=.267). Analyses of between network FC were highly similar to the main results with the full sample of boys with ADHD, although all *p*-values were slightly increased (Table S7). To further explore whether sex differences in inattention symptom severity are driving the observed sex differences in FC, we created high-and low-symptom severity groups within each diagnostic group and conducted a 2 Diagnosis x 2 Inattention Symptom Severity ANOVA for each fronto-subcortical pair, the results of which did not include any diagnosis x inattention symptom severity interactions (all *p*s > .469). Collectively, these analyses support our conclusion that the observed sex differences are not due to greater symptom severity among the ADHD girls but may in fact reflect a true sex difference.

| **Table S7.** Intrinsic functional connectivity of fronto-subcortical pairs for children with attention-deficit hyperactivity disorder (ADHD) and typically developing (TD) children in the full sample (n=147) and among a reduced sample including a subset of boys with ADHD (n=17/52) with similar inattention symptom severity T-scores as the sample of girls with ADHD (n=112). | | | | | | | | | | |
| --- | --- | --- | --- | --- | --- | --- | --- | --- | --- | --- |
|  | Full Sample (n=147) | | | | | Reduced Sample (n=112) | | | | |
|  | Dx | Dx×Sex | All | Girls | Boys | Dx | Dx×Sex | All | Girls | Boys |
|  | *p* | *p* | *d* | *d* | *d* | *p* | *p* | *d* | *d* | *d* |
| F1-S1 | .012* | .291 | 0.42* | 0.66^†^ | 0.27 | .054 | .153 | .39 | .72* | .11 |
| F2-S1 | .092 | .079 | 0.28 | 0.64^†^ | 0.01 | .168 | .050 | .28 | .70* | .12 |
| F3-S1 | .041^†^ | .048^†^ | 0.33^†^ | 0.74^†^ | 0.01 | .080 | .058 | .34 | .76* | .03 |
| F1-S2 | .002* | .042^†^ | 0.51* | 0.94* | 0.20 | .008* | .051 | .53* | .97* | .15 |
| F2-S2 | .183 | .172 | 0.22 | 0.49 | 0.01 | .186 | .279 | .27 | .50 | .05 |
| F3-S2 | .805 | .310 | 0.04 | 0.23 | 0.14 | .723 | .461 | .07 | .22 | .08 |
| Note. Statistical *p*- and *d*-values are based on the estimated marginal means. Cohen’s *d* is reported as an estimate of effect size.  *Significant effect after FDR correction applied for six tests; ^†^*p*<.05 without FDR correction. | | | | | | | | | | |

**S8. Correlations between fronto-subcortical FC and delay discounting across ADHD and TD groups.**

| **Table S8.** Correlation between FC of fronto-subcortical network pairs and delay discounting (area over the curve) across ADHD and TD groups. | | | | | | | |
| --- | --- | --- | --- | --- | --- | --- | --- |
|  | *n* | F1-S1 | F2-S1 | F3-S1 | F1-S2 | F2-S2 | F3-S2 |
| Money Discounting | 147 | .118 | .111 | -.235* | .031 | .044 | -.027 |
| Gametime Discounting | 137 | -.061 | .028 | -.141 | -.014 | .125 | -.133 |
| Note. Pearson’s correlation coefficient reported above. **p* < .01, survives FDR correction for 12 tests. | | | | | | | |
